# The intrepid urban coyote: a comparison of bold and exploratory behavior in coyotes from urban and rural environments

**DOI:** 10.1038/s41598-019-38543-5

**Published:** 2019-02-14

**Authors:** Stewart W. Breck, Sharon A. Poessel, Peter Mahoney, Julie K. Young

**Affiliations:** 1 USDA-WS-National Wildlife Research Center, 4101 Laporte Ave., Fort Collins, CO 80521 USA; 2 Department of Wildland Resources, Utah State University, 5230 Old Main Hill, Logan, UT 84322 USA; 3 Present Address: U.S. Geological Survey, Forest and Rangeland Ecosystem Science Center, 970 S. Lusk St., Boise, Idaho 83706 USA; 4 Present Address: School of Environmental and Forest Sciences, University of Washington, Seattle, WA 98195 USA; 5 USDA-WS-National Wildlife Research Center-Predator Research Facility, Millville, UT 84326 USA

## Abstract

Coyotes (*Canis latrans*) are highly adaptable, medium-sized carnivores that now inhabit nearly every large city in the United States and Canada. To help understand how coyotes have adapted to living in urban environments, we compared two ecologically and evolutionarily important behavioral traits (i.e., bold-shy and exploration-avoidance behavior) in two contrasting environments (i.e., rural and urban). Boldness is an individual’s reaction to a risky situation and exploration is an individual’s willingness to explore novel situations. Our results from both tests indicate that urban coyotes are bolder and more exploratory than rural coyotes and that within both populations there are individuals that vary across both spectrums. Bolder behavior in urban coyotes emerged over several decades and we speculate on possible processes (e.g., learning and selection) and site differences that could be playing a role in this behavioral adaptation. We hypothesize that an important factor is how people treat coyotes; in the rural area coyotes were regularly persecuted whereas in the urban area coyotes were rarely persecuted and sometimes positively rewarded to be in close proximity of people. Negative consequences of this behavioral adaptation are coyotes that become bold enough to occasionally prey on pets or attack humans.

## Introduction

Humans are altering landscapes throughout the world, and understanding how species adapt to altered environments and what can be done to enhance coexistence are important endeavors to maintain species across these new landscapes. Urban environments (i.e., cities and suburban areas) are one of the more dramatic forms of human alteration to terrestrial environments and generally result in loss of biodiversity^1–3^. However, some wildlife species are capable of adapting to urban environments, resulting in robust populations of a variety of bird and mammal species^4–6^.

In an effort to understand why certain species are better at adapting to the challenges of urban environments, a primary research focus has been on evaluating behavioral adjustments made by animals^5,7,8^. Of particular interest has been how animal behavior differs in contrasting landscapes (e.g., rural, urban, and natural systems), with key findings indicating that animals generally become more aggressive, exploratory, and bold in urban environments compared to animals in rural and natural systems^6^. Distinctions between these behaviors are important. Thus, aggressive behavior is defined as an individual’s agonistic reaction toward conspecifics; exploratory behavior is an individual’s reaction to a novel food, habitat, or object; and boldness is an individual’s reaction to a risky situation like encountering a predator or interacting with humans^9^. Understanding how these behaviors change in different environments will be particularly important for larger carnivore species that often must coexist with people on human modified landscapes and where the development of bold or aggressive behavior could create difficult human-wildlife interactions.

The development of bolder, more aggressive, and more exploratory urban individuals in a wide variety of species would imply that these behavioral traits are necessary or confer advantages in urban areas. Shifts in behavior by animals to new environments are believed to be a response to an altered landscape where animals are adapting to a variety of new challenges like a modified sensory environment, disruption of physiological processes, changes in habitat characteristics, creation of novel food sources, and alterations in species interactions^5,10^. Of these factors, alterations in predation pressure has received the most attention; researchers have demonstrated repeatedly that such alterations can modify aggressive, bold, and exploratory behavior in animals^11–13^.

In addition to understanding what factors drive behavioral change, there is also an emphasis on understanding the mechanisms of change. Generally, three distinct mechanisms could be operating: (1) learning, whereby individuals have inherent behavioral plasticity that allows them to adjust their behavior based on environmental feedback; (2) sorting, whereby only individuals with appropriate behaviors are able to colonize urban environments; and (3) selection, an evolutionary response implying genetic selection of particular traits^5^. To date most evidence indicates that changes in behavior are a result of individual phenotypic plasticity and the ability to learn behavior that matches the requirement of the urban environment^5,14^, but it is possible that all three processes act in concert.

For carnivore species, little work has addressed questions of bold and exploratory behavior in contrasting environments or what factors drive behavioral change. Notable exceptions are the work of Greenberg *et al*.^15^, who identified predation by lions (*Panthera leo*) as a driving factor in altering boldness and other behavioral traits in juvenile spotted hyenas (*Crocuta crocuta*), and Wheat and Wilmers^16^ and Benazzo^17^, who indicated that shy brown bears (*Ursus arctos*) in Europe are the result of a long history of human persecution.

Our primary research objective was to compare bold and exploratory behavior of rural and urban coyotes (*Canis latrans)*. The impetus for comparing these behaviors was the emergence of extreme forms of human-coyote conflict (i.e., coyotes attacking and preying on pets and occasionally attacking people) in our urban study area^18^, prompting resource managers to ask if coyotes had become bolder. We compared behavior between rural and urban coyotes because it provides a means for understanding how behavior has changed. In our study system, bold behavior leading to extreme forms of conflict emerged several decades after colonization, implying that the process of behavioral change was not immediate. Our secondary objective was to speculate on processes and external factors that may be playing a role in altering behavior in an effort to help guide future research investigating causal mechanisms.

This research is relevant because every major city in the continental United States has been colonized by coyotes^19^ and many experience similar types of conflict^20–22^. Furthermore, other mid-sized and larger carnivores (e.g., dingos [*Canis lupus dingo*], golden jackals [*Canis aureus*], spotted hyenas, and leopards [*Panthera pardus*]) are showing similar abilities to adapt to urban environments^23–26^. Thus, we predicted that urban coyotes would be bolder and more exploratory relative to rural coyotes, a prediction first proposed by Baker and Timm^20^ and Timm *et al*.^21^. We did not explicitly address aggressive behavior (i.e., reaction to conspecifics) in coyotes but note that the level of bold, aggressive, and exploratory behavior expressed by an individual are often correlated^27^.

Following criteria provided by Reale *et al*.^9^ and Smith and Blumstein^28^, we used two behavioral tests (flight initiation distance [FID] and a novel object test) to measure bold and exploratory behavior in two distinct populations of coyotes. FID is the distance at which an animal begins to flee from an approaching predator or threat^29,30^, and novel object tests use new objects in the environment to measure the willingness of an individual to take risk and approach^27,31–33^. Young *et al*.^34^ used both FID and novel object tests on captive coyotes and recommended both tests would be useful for understanding how bold and exploratory behavior varies across coyote populations. We conducted our work at a rural area (central Utah, USA) and an urban area (Denver, Colorado, USA) and details of each area can be found in Mahoney *et al*.^35^ and Poessel *et al*.^36^.

## Results

### Flight Initiation Distance

**Figure 1:**
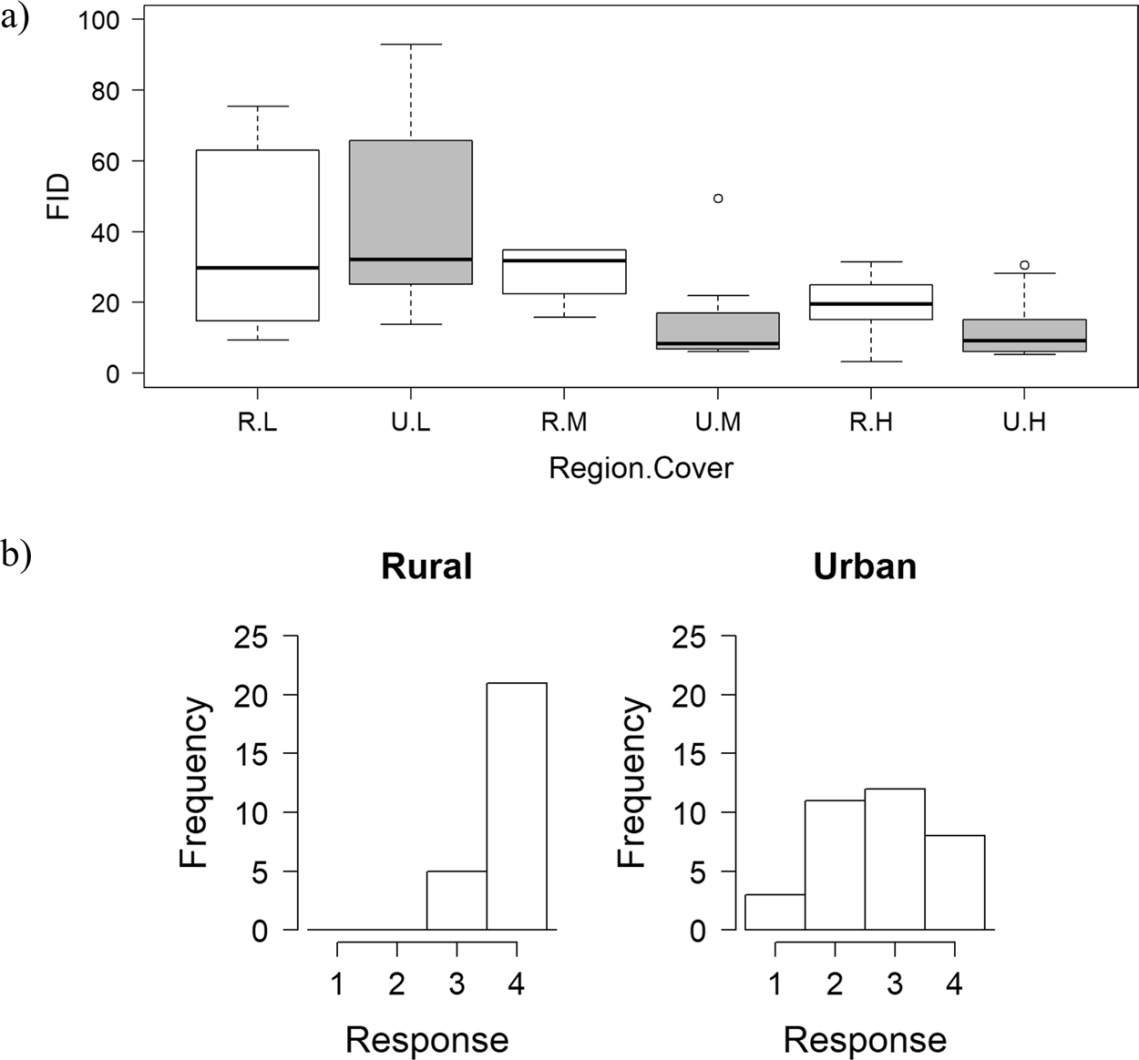
Results of Flight Initiation Distance (FID) tests on coyotes: (**a**) boxplots [median value = bold horizontal line, 1^st^ and 3^rd^ quartiles = bottom and top of box, respectively; whiskers = the most extreme data point that is no more than 1.5 times the length of the box; and outliers = circles] of coyote FID for rural (R) and urban (U) areas (Region) and amount of vegetative Cover (L = low, M = medium, and H = high) and (**b**) the frequency of the behavior response of coyotes during the FID test. See Table 4 for definition of behavioral responses 1–4.

With the FID test we measured two coyote reactions: distance at which coyotes fled an approaching human and behavioral reaction while fleeing. For the distance data we attained 23 measurements from 14 radiocollared individuals in the rural area and 39 measurements from 17 radiocollared individuals in the urban area. Because we had repeated measurements on 61% of the known individuals, we estimated repeatability (Nakagawa and Schielzeth^37^, Stoffel and Nakagawa.^38^) of distance measurements and found that individuals did not show consistency in behavior (*R* = 0.14, SE = 0.15, CI = 0.00–0.51). Our modeling results strongly indicated that cover was an important factor influencing distance and weakly indicated that rural coyotes would initiate flight at greater distances than urban coyotes. The top model with the most weight (0.64) had only cover as the explanatory variable and the next two ranked models (combined model weight = 0.36) included both cover and region, indicating flight distance varied by cover type (Table 1: Flight Distance). In two of the three top models, the coefficient values for the variable “region” had confidence intervals that overlapped zero (Supplementary Table S1), further indicating that the difference between coyotes in rural and urban areas was weak. The distance at which coyotes fled was somewhat greater for rural coyotes in medium and high cover types but not in low cover types (Fig. 1a). The importance of cover shows how variation in flight distance increases substantially in situations with low cover for both rural and urban coyotes (Fig. 1a).

**Table 1:** Modeling results of two flight initiation distance tests: flight distance and behavioral state. In the Model Name, region indicates either rural or urban; cover indicates either low, medium, or high; null is the model with no fixed effects; “+” indicates an additive effect; and “*” indicates an interaction effect. K is the number of parameters. AICc is the small sample size Akaike’s Information Criterion value of the model. ΔAICc is a measure of each model relative to the model with the lowest AICc value; Weight is the Akaike weight of each model varying from 0–1 and provides a measure of the strength of evidence for that model relative to the other models; and LL is the log likelihood value of the model.

Data Type:	Model Name	K	Δ AICc	Weight	LL
Flight Distance	Cover	5	0.0	0.64	−259.5
	Region + Cover	6	1.8	0.26	−259.2
	Region * Cover	8	3.7	0.10	−257.6
	Null	3	19.7	0.00	−271.7
	Region	4	20.5	0.00	−271.0
Behavioral State	Region + Cover	7	0.0	0.70	−54.4
	Region	5	1.7	0.30	−57.8
	Null	4	22.0	0.00	−69.1
	Cover	6	24.8	0.00	−68.1
	Region * Cover	Interaction model not supported

For the behavioral state data, we attained 26 measurements from 15 coyotes in the rural study area; none reacted with a low-level response (level 1 or 2, Fig. 1b), and most measurements (81%) showed a level 4 response (i.e., coyote quickly fled the area and did not stop or look back). In contrast, we attained 34 measurements from 17 urban coyotes, and 41% of measurements were level 1 or 2 responses (Fig. 1b) where individuals moved slowly away, stopped, and looked back as they retreated, sometimes within 3 m of the original starting point. We attained multiple measurements on 69% of coyotes and found that repeatability of reaction measurements showed little individual consistency (*R* = 0.12, SE = 0.043, CI = 0.00–0.16). Our modeling results supported that urban coyotes exhibited bolder behavior when reacting to an approaching person. The 2 models with model weight >0.99 had region as a covariate (Table 1: Behavioral State), and in the top model the region coefficient had a confidence interval that did not overlap zero (Supplementary Table S2), indicating meaningful differences in behavior between rural and urban coyotes.

### Novel Object Test

For the novel object test, we used an attractant at baseline sites to record baseline behavior and compared this to our treatment where a novel object was placed around the attractant. We measured three distinct coyote responses, number of visits to sites, distance to attractant, and behavioral reaction. For the visit data, two models accounted for all the support (Table 2: Visits to Sites) indicating that both trial (baseline vs. treatment) and region (rural vs. urban) were important variables. Home ranges of coyotes were approximately 3 times larger in the rural versus urban study areas^36,39^; thus, we expected the urban sites would have more visits than the rural sites at both baseline and treatment sites. The second ranked model supported an interaction between trial and region (AICc weight = 0.40, Table 2: Visits to Sites), indicating that the effect of the novel object was greater in the rural environment. To demonstrate this interaction, we calculated the ratio of total visits (rural/urban, Table 3) between the baseline sites (12/96 = 0.13) and treatment sites (1/25 = 0.04). This calculation shows that the effect of the novel object treatment was over 3 times stronger on rural coyotes compared to urban coyotes, providing evidence that the novel object reduced visitation to sites for coyotes in the rural area. However, it should be noted that this effect is only weakly supported as indicated by the fact that that the confidence interval for the interaction coefficient overlapped zero (Supplementary Table S3).

**Table 2:** Modeling results of three tests of the novel object data: visits to sites, spatial response, and behavioral response. For the Model Name, region is either rural or urban; trial is either baseline or treatment; distance is either far, close, or on; behavior is either vigilant, investigative, or comfort; null is the model with no fixed effects; “+” indicates an additive effect; and “*” indicates an interaction effect. K is the number of parameters. AICc is the small sample size Akaike’s Information Criterion value of the model. ΔAICc is a measure of each model relative to the model with the lowest AICc value; Weight is the Akaike weight of each model varying from 0–1 and provides a measure of the strength of evidence for that model; LL is the log likelihood value of the model.

Data Type:	Model Name	K	ΔAICc	Weight	LL
Visits to Sites	Region + Trial	3	0.0	0.60	−168.89
	Region*Trial	4	0.8	0.40	−168.13
	Region	2	51.7	0.00	−195.85
	Trial	2	98.2	0.00	−219.10
	Null	1	149.9	0.00	−246.05
Spatial Response	Region*Trial + Distance	13	0.0	0.87	−1247.2
	Region + Trial + Distance	11	3.8	0.13	−1250.1
	Region + Distance	9	13.0	0.00	−1255.7
	Distance	7	32.8	0.00	−1266.7
	Region + Trial	7	52.3	0.00	−1276.4
	Trial + Distance	9	61.7	0.00	−1281.1
	Region	5	62.6	0.00	−1282.6
	Trial	5	65.7	0.00	−1284.2
	Null	3	76.3	0.00	−1290.5
Behavioral Response	Region + Trial + Behavior	11	0.0	0.67	−1218.2
	Region + Behavior	9	2.3	0.21	−1221.0
	Behavior	7	4.7	0.06	−1223.3
	Trial + Behavior	9	5.0	0.06	−1222.4
	Region*Trial + Behavior	13	12.6	0.00	−1224.1
	Region	5	35.6	0.00	−1239.7
	Null	3	41.9	0.00	−1243.9
	Trial	5	42.1	0.00	−1243.0
	Region + Trial	7	108.7	0.00	−1276.3

**Table 3:** Number of visits recorded by coyotes investigating an olfactory attractant (Baseline) and an olfactory attractant surrounded by a novel object (Novel Object). Each region (rural and urban) had 30 sites (15 each with the baseline and novel object), and we recorded the number of sites visited by coyotes and the total number of visits by coyotes.

Region: [Treatment]	Number of Sites	Number of Sites Visited by Coyotes	Total Visits by Coyotes
Rural: [Novel Object]	15	1	1
Rural: [Baseline]	15	6	12
Urban: [Novel Object]	15	7	25
Urban: [Baseline]	15	8	96

**Figure 2:**
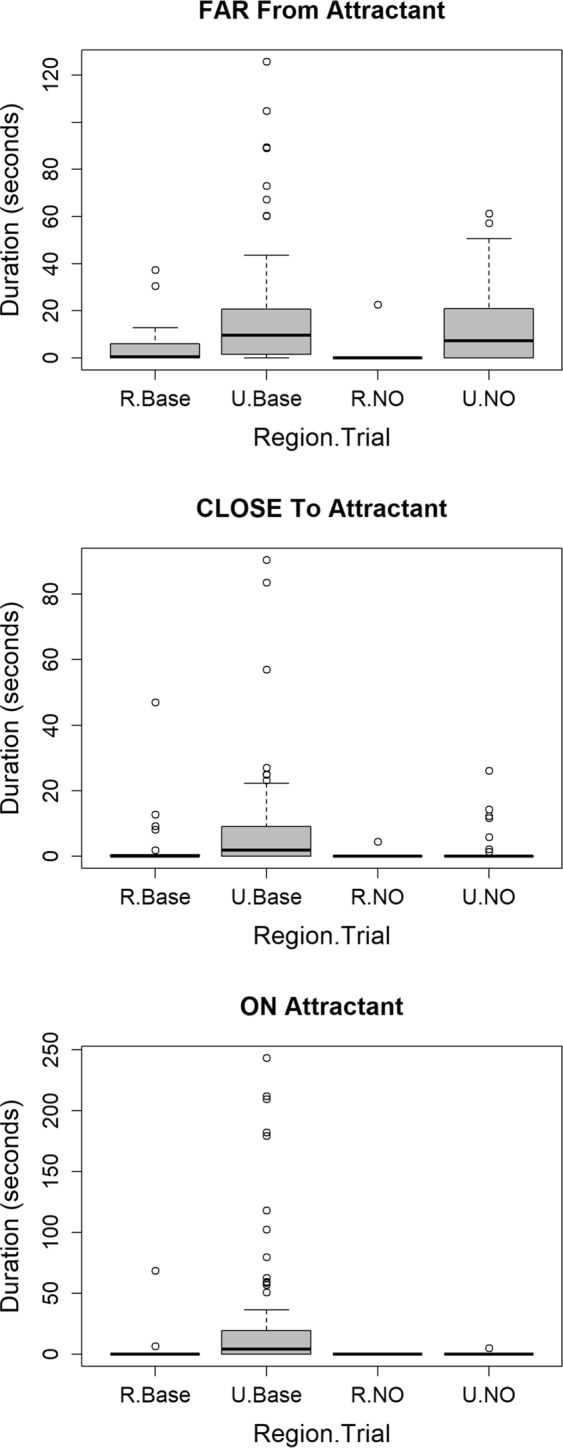
Boxplots [median value = bold horizontal line; 1^st^ and 3^rd^ quartiles = bottom and top of box, respectively; whiskers = the most extreme data point that is no more than 1.5 times the length of the box; and outliers = circles] of the amount of time coyotes in the rural (R) and urban (U) areas (Region) spent at three distances (far, close and on) during the novel objects test at baseline (Base) and treatment (NO) sites (Trial).

For the distance response, two models had ΔAICc values <4 and the most complex model had 87% of the support (Table 2: Spatial Response), indicating that the amount of time coyotes spent at different distances to the novel object (far, close, on) differed between rural and urban sites and by trial (baseline vs. treatment). At all three distances, urban coyotes spent more time at the baseline sites than rural coyotes (Fig. 2). At the far distance, urban coyotes spent more time at the novel object than rural coyotes, but this difference faded at the closer distances (close and on), indicating an interaction between trial and region was supported (Table 2 and Supplementary Table S4). Altogether, these results provided evidence that the spatial response of coyotes was stronger and less exploratory in the rural area. Finally, we also found a great deal of individual variation in both rural and urban areas for all three spatial responses (Fig. 2).

**Figure 3:**
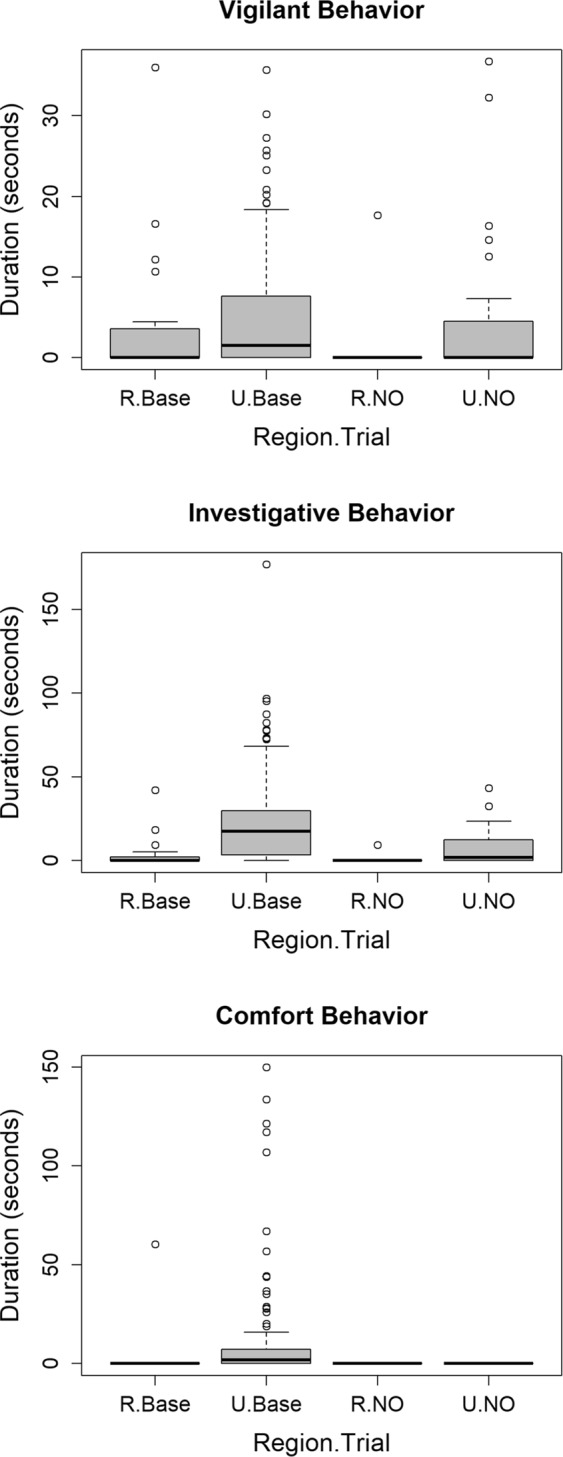
Boxplots [median value = bold horizontal line; 1^st^ and 3^rd^ quartiles = bottom and top of box, respectively; whiskers = the most extreme data point that is no more than 1.5 times the length of the box; and outliers = circles] of the amount of time coyotes in the rural (R) and urban (U) areas (Region) spent in three behavioral responses (investigative, vigilant, and comfort) during the novel objects test at baseline (Base) and treatment (NO) sites (Trial).

For the behavioral state analysis, two models had ΔAICc values <4 (Table 2: Behavioral Response), and the model with the greatest support (weight = 0.67) indicated that the amount of time coyotes exhibited a particular behavior varied depending on the behavioral response (vigilant, investigative, or comfortable), region (rural vs. urban), and trial (baseline vs. treatment) (Supplementary Table S5). Urban coyotes spent more time investigating and being vigilant than rural coyotes for both the baseline and novel object treatment and more time displaying comfort behavior than rural coyotes at baseline sites (Fig. 3). With the novel object present, no coyotes displayed any comfort behavior at either urban or rural sites and only a single coyote displayed any comfort behavior at the rural baseline site, whereas comfort behavior was relatively common at the urban baseline site. Similar to the distance data, we found a great deal of individual variation within rural and urban coyotes and for all three types of behavior (Fig. 3).

## Discussion

We found strong evidence that coyotes were bolder and more exploratory in our urban study area than our rural study area, results similar to many studies addressing differences in animal behavior between rural and urban landscapes^6^. The reaction of coyotes to the FID test revealed that 46% of coyotes in urban areas showed a relatively low-level flight response to an approaching human (level 1 and 2). In contrast, the vast majority (80%) of coyotes in rural areas showed the strongest flight response (level 4) in which individuals fled rapidly without looking back. These results are similar to another study that measured how urban coyotes responded to hazing and found that a few coyotes would even approach people as they were attempting to scare or haze the coyotes^40^.

Our other measurement from the FID test (i.e., distance at which a coyote initiated flight, Fig. 1a) was somewhat ambiguous about whether coyotes were bolder in our urban study area. The distance at which animals initiate flight should decrease with bolder individuals^29,30,41^, thus we expected urban coyotes to have initiated flight at shorter distances. We saw evidence of this pattern in medium and high levels of vegetation cover (Fig. 1a), but overall there was high variation in our data, particularly when coyotes were in low density cover that limited statistical difference between the rural and urban regions. More importantly, we believe our method of calculating flight distance may not adequately measure bold behavior. Our measurements of flight distance were taken only while coyotes were stationary (presumably bedded down), whereas most FID studies take measurements while animals are foraging and active. While bedded down, coyotes may be altering their flight behavior and using a strategy to elude rather than flee from a predator. Our flight distance measurements may be illustrating this alternative strategy for reacting to predators and therefore confound our results. Of particular note is the high variation in flight distance seen in the low cover types in both rural and urban areas (Fig. 1a). We speculate that deciding to flee an approaching human while hiding is likely a decision influenced by how secure an animal feels. It appears that coyotes in low cover have greater uncertainty about whether to flee or try to stay hidden, and this may reflect whether or not a coyote perceives it has been detected during the approach. We note that we initially tried to measure flight distance while animals were active, but in the rural area we found it logistically infeasible to observe coyotes without them detecting us and immediately running away at any distance.

Miranda *et al*.^6^ cautioned that interpreting behavior of urban adapted species solely on FID measurements risks confounding bold, aggressive, and risk-taking behaviors with habituation of animals to humans. They indicate that novel object tests offer a more robust mechanism for assessing behavioral changes in urban adapted species. Our results from the novel object test complement the FID results by indicating that urban coyotes are more willing to explore and take more risk. Urban coyotes had higher visitation rates, spent more time in close proximity to the novel object, and spent more time demonstrating investigative, vigilant, and comfort behaviors than rural coyotes, and this was true for both baseline and treatment sites. Of particular note is the fact that in the rural area, only a single coyote visited a site containing the novel object treatment, indicating that the novel object had a much stronger impact on how many sites were visited, how close coyotes approached the novel object, and the type of behavioral response coyotes had to the novel object. These results support the notion that urban coyotes are more likely or willing to explore novel environments, and we speculate below on external factors from the urban environment that may impact this behavior.

Several observations from our study provide an opportunity to infer which processes (sorting, selection, and learning) may play a role in the population level change in behavior that we documented. Results from our experiments demonstrated that both urban and rural populations of coyotes have individuals expressing bold/exploratory behavior (i.e., outlier observations in Figs 1, 2 and 3) and shy/avoidance behavior (i.e., prevalence of zeroes in data) and that repeatability of behavior within individuals was low. A sorting process would require consistent behavior within individuals and result in an urban population with a lower limit of shy behavior that is higher than that of the rural population. Our results do not show either of these patterns and thus do not support the idea of sorting as a process leading to bolder urban coyotes. The lack of repeatability does suggest that coyote behavior is quite plastic and lends support to the idea that learning plays a role in allowing coyotes to adapt to new environmental circumstances.

Another observation that allows us to speculate about mechanisms is that reports of conflict in the urban area did not appear until several decades after colonization^18^. This is relevant because conflict represents more extreme forms of exploratory behavior (e.g., coyotes getting into fenced yards to prey on pets), bold behavior (e.g., coyotes attacking people), and aggressive behavior (e.g., coyotes attacking dogs on leashes). The emergence of conflict decades after colonization implies that behavior change in urban coyotes was gradual thus arguing against sorting, which would result in rapid behavioral change, and supporting both learning and selection as possible mechanisms. Alternatively, slow emergence of conflict could imply that the number of conflicts increased along with the coyote population size, which increased gradually after colonization. This is an important alternative to consider as it implies that conflict has little to do with behavior and instead is linked with coyote population size. Better understanding of coyote population dynamics relative to levels of conflict would be necessary to address this hypothesis; information we did not have from our system.

Other research on coyotes also demonstrates plasticity in coyote behavior^42^ and importantly indicates that learning and not selection is the more important factor affecting behavioral change^43^. From a management perspective, understanding which mechanism is driving the changes in behavior is important because individuals that are extremely bold and aggressive are likely responsible for the more serious conflicts with humans and pets^44^. If the process is driven primarily by learning, then implementing techniques that teach coyotes to fear humans could play an important role in reducing conflict.

Regardless of the mechanisms, our results indicate that there are important differences between urban and rural environments that allow urban coyotes to become bolder and more exploratory. At a broader scale, there is a great deal of research that supports predation^11,15^ and specifically human predation^45–49^ as an important factor driving a variety of behavioral changes (including bold, aggressive and exploratory behavior) in wildlife populations. In our study systems, there are stark contrasts in how people behave as predators towards coyotes. In our rural study area, coyotes are regularly trapped and shot by hunters, trappers and wildlife managers^35^, whereas in our urban area, purposeful human persecution is rare. It is easy to speculate how such human pressure in rural areas could suppress bold and exploratory behavior, but caution is merited with this explanation. In our rural study area, mountain lions also acted as predators of coyotes and this predation could act to suppress bold and exploratory behavior. Furthermore, in the urban area there are additional anthropogenic activities that may encourage more exploratory and bold behavior in coyotes. For example, purposeful feeding of coyotes is known to occur, though this is hard to quantify; people also inadvertently make a variety of anthropogenic food sources available, which could encourage more exploratory behavior; and urban residents often display disregard and sometimes even fear toward coyotes^40^, which may influence learning and the development of bold behavior toward humans. Finally, the urban environment contains a variety of pollutants and toxicants (e.g., rodenticides^50^) that are likely not present in most rural environments, and it is unknown how such contaminants may directly or indirectly influence behavior.

We speculate that the primary factor influencing the adaptive changes in coyote behavior is human behavior. Our conclusion that bold and exploratory behaviors are suppressed by humans hunting and trapping coyotes and encouraged by urban human behavior agrees with the hypothesis that neophobia (fear of novel things) increases in more dangerous habitats^6^. However, more research from other systems is necessary to support or refute the generality of our results as well as elaborate on the mechanisms driving change, not only in coyotes but other species of carnivores as well. For example, comparing bold and exploratory behavior of coyotes in national parks where hunting is not allowed and human development is sparse to behavior of coyotes in environments where hunting is allowed would help elaborate whether human persecution is a driving factor versus other factors like the availability of novel anthropogenic food. From a conservation perspective, elaborating on how bold, exploratory, and aggressive behavior changes in carnivore species and the role that humans play will help inform management strategies as societies around the world learn to coexist with these species.

## Methods

### Flight Initiation Distance

We used animals captured and radio-collared to perform the FID (see Poessel *et al*.^36^ for a description of the handling of urban coyotes and Mahoney *et al*.^35^ for a description of handling rural coyotes). All FID work was done during daylight hours as opportunities allowed. The general strategy was to use radio-telemetry equipment to locate an individual during a time when it was stationary (i.e., presumably bedded down) and from a distance that would not disturb it. If there was any sign that the animal was moving during this phase and/or the individual was located on private property, then the trial was terminated unless access could be obtained. Once an individual was in a suitable location, we then approached. During the approach we walked in a straight line toward the coyote and watched the area where the coyote was believed to be bedded down. When the coyote began to move, we measured the distance to that point from the point where we were standing at the time (flight distance). We also measured the response of the coyote on a scale of 1–4 with 1 being the mildest response and 4 being the strongest response (defined in Table 4; behavioral state). After the trial was performed, we then recorded the density of cover from which the coyote fled as either low (where the coyote was not hidden, was out in the open, and was seen upon approach), medium (where the coyote was hidden by sparse vegetation and could be seen upon closer approach), or high (where the coyote was completely hidden and was not seen upon approach). We note that when performing these trials in high cover, it was often difficult to see the coyote and for some of these trials, we relied on visual and auditory indications that the individual flushed and then estimated the place from which it flushed. We attempted to perform 3 separate trials on each radio-collared individual, where trials were separated by at least a week of time. Measurements were taken in both rural and urban areas from mid-February through mid-November 2013 with 69% of observations collected during June–August.

**Table 4:** Response coding of coyote reaction (i.e., Behavioral State) to the flight initiation distance test performed on coyotes in both rural and urban study sites.

Rank	Description
1	Coyote moves less than 3 m away after input and stops and looks back in the direction of the stimulus less than 3 m from the original starting point.
2	Coyote moves more than 3 m away after input and stops and looks back in the direction of the stimulus more than 3 m away from the original starting point.
3	Coyote moves away from the area, either quickly or slowly, and looks back while retreating.
4	Coyote flees the area after input. Locomotion involves rapid directed movement. Coyote does not stop or look back as it retreats.

We analyzed the flight distance and behavioral state data separately (Table 1). For the flight distance analysis, we used distance as a continuous response variable and used linear mixed modeling (package “lme4” in R^51^) to determine if the flight distance was different between regions (rural vs. urban) and/or cover (low, medium, or high). We developed five models (Table 1) that tested for various combinations of these fixed effects, including a null model (i.e., none of the covariates). In all models we treated coyote ID as a random effect to account for the repeated observations on most of the individuals.

For the behavioral state data, our response was ordinal (i.e., 1, 2, 3, or 4), and therefore we used ordered logistic regression (package “ordinal” in R^52^) and tested for differences in response between regions and cover. We developed five models (Table 1) that tested for various combinations of these fixed effects, including a null model (i.e., none of the covariates). We were unable to run a model testing for an interaction between region and cover because the rural area had no data for the two lowest response categories. For both analyses, we determined the importance of each fixed effect by using Akaike’s Information Criterion corrected for small sample size (AICc) values to rank models^53–55^ and by investigating the confidence intervals of the coefficient values for all fixed effects in supported models.

We also performed a repeatability analysis^37^ on the flight distance data and behavioral state data (package “rptR” in R^38^). Because the flight distance was right skewed, we first transformed the response variable by taking the square root. We then used the rpt function and controlled for fixed effects by including region and cover in the model, used a Gaussian data type, and performed 1000 parametric bootstrap iterations. For the behavioral state data, we performed the same analysis as described above except we treated the ordinal data as non-Gaussian and used a Poisson data type.

### Novel Object Test

We identified 30 sites in both rural and urban study areas to perform the novel object test. To ensure independence between sites, we used the average home range size of coyotes in each study area^36,39^ to determine a minimum distance between sites. At each site we found a single location to set up the equipment used to elicit and measure coyote responses. At the 30 urban sites, we concentrated on finding locations that were secluded within open spaces, greenbelts, and parks that we believed had a high probability of coyotes visiting. At the 30 rural sites, we chose locations that were away from roads and major trails to reduce the amount of human disturbance. At all locations we hammered a t-post in the ground so that a camera could be mounted on the post at ~1.2 m above the ground. We put in t-posts at least 3 weeks prior to any trials starting so coyotes could habituate to the posts. We then randomly selected half the sites in each study area to be baseline without a novel object and half to be novel object treatments.

At all sites, we dug a small hole in the ground (~50 mm in diameter and 50 mm deep) ~3.5 m from the t-post. We placed a heaping tablespoon of meat bait (Sweet Meat Predator Bait, Russ Carman, New Milford, Pennsylvania) in the hole and then stuffed grass into the hole. On top of the grass we placed a fatty acid tab (plaster disc ~25 mm diameter that is impregnated with a fatty acid scent, Pocatello Supply Depot, Idaho), which is known to attract a variety of carnivores including coyotes. We refer to the combination of the meat bait and the fatty acid tab as the attractant, and this was the only object placed at the baseline sites.

At the treatment sites we then established a visual novel object that surrounded the attractant. To do this, we hammered in 4 wooden stakes so that the stakes formed a 1 m^2^ area around the attractant. We tied a rope at the top of the stakes ~1 m above the ground. At all sites, we then placed a Bushnell 8.0 megapixel Trophy HD camera (Bushnell Outdoor Products, Overland Park, Kansas) capable of recording video on the t-post so that it was pointing directly at the objects. We only had 6 cameras at each study area, so we matched 3 baseline and 3 treatment sites at each study area until we had worked through all of the sites. We sometimes recorded multiple videos of coyotes at a site and considered videos as independent if at least 10 minutes elapsed between videos.

To analyze the novel object data, we extracted data from each video on three separate variables: number of coyote visits per site, distance of coyote from the attractant, and behavioral reaction to the objects. To tabulate behavior from video footage, we used the Noldus Observer XT (2013, Wageningen, The Netherlands) event logging software. We created a coding scheme to capture all tangible behaviors coyotes displayed that were associated with investigation of the attractant or novel object and scored each video continuously.

For the visit data, we counted the number of sites visited and the number of visits per site under the premise that shyer individuals would avoid the attractant or novel object, and thus it would be difficult to measure their behavior. However, a fundamental confounding problem with these count data was that rural coyote home ranges were approximately 3 times larger than urban coyote home ranges^36,39^. Thus, we expected that counts of sites visited and number of visits per site would be inherently lower at the rural site. However, by testing for an interaction between region (urban vs. rural) and trial (baseline vs. treatment), we were able to evaluate whether coyotes in the rural area reacted more strongly to the attractant or novel object than did urban coyotes. We used Poisson regression to analyze the visit data (response variable = number of visits per site) and ran 5 competing models (Table 2: Visits to Sites) that investigated whether counts varied by region and trial.

From each video of coyotes, we also quantified the amount of time spent by the coyote within one of three distance classes from the attractant: (1) far: coyote stayed at least one body length from the attractant; (2) close: coyote was within one body length of the attractant; and (3) on: coyote made contact with the attractant with its nose, paw, or body. To analyze these data, we converted the response variable (time) to a count (based on seconds) by discretizing the data and used a zero-inflated negative binomial mixed model (package “glmmADMB” in R^56^). We ran 9 competing models (Table 2: Spatial Response) with region, trial, and distance (far, close, and on) as fixed effects. Because we could rarely identify coyotes as individuals in the videos, it is likely that sometimes there were multiple videos of the same individual. Treating these observations as independent would not be valid. To address this issue, we accounted for the likely repeated measurements by using a mixed-effects model which allows handling non-independent data due to group membership by including each independent coyote video nested within each site as a random effect.

The final data extracted from the video was a behavioral reaction, which involved quantifying the amount of time spent as vigilant, investigating, or comfortable. We defined vigilant as behavior indicating caution or apprehension and categorized coyotes as vigilant if their tail was tucked, they walked hesitantly toward the object, flinched, paced around the object, or assumed a crouched position. We defined investigating as behavior indicating the coyote was not concerned about the attractant or novel object, which included a tall posture, an erect tail, directed travel toward the object without pausing, and a relaxed stature. We defined comfortable as the time spent performing any of the following behaviors: shaking, rolling, urinating, defecating, digging, pawing, scratching, stretching, eating, or taking the bait. A few animals also showed a behavior of jumping and running away, but we did not include this in the analysis because it constituted about 12 seconds of data (0.09%) and was evenly distributed between rural and urban areas. We quantified each of these three behaviors for every individual. Similar to the distance data, we discretized the response variable (time) and used zero-inflated negative binomial mixed modeling with each coyote video nested within each site as a random effect and region, trial, and behavioral state (investigating, vigilant, or comfortable) as fixed effects. We used a random effect in the mixed model to account for the possibility of an individual coyote being recorded in repeated videos. We developed 9 competing models (Table 2: Behavioral Response) that tested for various combinations of the fixed effects, including a null model (i.e., none of the covariates). For all 3 data types from the novel object test, we derived inference from our top models and coefficient values as described above.

### Ethical Approval and Informed Consent

Approval to undertake this project was granted by the USDA Institutional Animal Care and Use Committee (National Wildlife Research Center IACUC; QA-1972 and QA-1907) and Utah State University IACUC (#2182). The project was conducted in accordance with the guidelines and regulations of these approvals.

## Supplementary information

Supplementary Table

## Data Availability

The datasets generated and/or analyzed during the current study are available from the corresponding author on reasonable request.
