# Supplementary material for: The intrepid urban coyote: a comparison of bold and exploratory behavior in coyotes from urban and rural environments
Source: Sci Rep. 2019 Feb 14;9:2104. doi: 10.1038/s41598-019-38543-5 (PMC6376053; doi:10.1038/s41598-019-38543-5)
Supplement: Supplementary file 1 — Supplementary Table [file 41598_2019_38543_MOESM1_ESM.pdf]

The intrepid urban coyote: a comparison of bold and exploratory behavior in coyotes from urban and rural environments

Stewart W. Breck[1*], Sharon Poessel[2,3], Peter Mahoney[2,4], Julie K. Young[2,5]

Supplementary Table S1. Coefficient values (Beta), standard error (SE), lower confidence level (LCL), and upper confidence level (UCL) for all fixed effects in models with a ∆ AICc score < 4 from Table 1: Flight Distance. Shaded rows represent coefficient values in which the confidence interval does not overlap zero, an indication of the significance of this value.

**Model Rank and Name**	**Fixed Effect**	**Beta**	**SE**	**LCL**	**UCL**
Model Rank = 1	Cover Low	25.40	4.68	16.26	34.61
Cover	Cover Med.	6.93	5.59	-4.01	17.89
Model Rank = 2	Region	-3.47	4.40	-12.10	5.16
Region + Cover	Cover Low	24.94	4.70	15.74	34.14
	Cover Med.	6.71	5.56	-4.19	17.62
Model Rank = 3	Region	-7.68	5.95	-19.36	3.99
Region * Cover	Cover Low	16.39	7.15	2.38	30.40
	Cover Med.	8.67	9.15	-9.27	26.61
	Region:Cover Low	14.86	9.27	-3.31	33.02
	Region:Cover Med.	-3.97	11.34	-26.20	22.23

Supplementary Table S2. Coefficient values (Beta), standard error (SE), lower confidence level (LCL), and upper confidence level (UCL) for all fixed effects in models with a ∆ AICc score < 4 from Table 1: Behavioral State. Shaded rows represent coefficient values in which the confidence interval does not overlap zero, an indication of the significance of this value.

**Model Rank and Name**	**Fixed Effect**	**Beta**	**SE**	**LCL**	**UCL**
Model Rank = 1 Region + Cover	Region	-3.25	0.70	-4.62	-1.88
	Cover Low	-1.58	0.63	-2.81	-0.35
	Cover Med.	-0.89	0.94	-2.73	0.95
Model Rank = 2 Region	Region	-2.79	0.64	-0.61	1.89

Supplementary Table S3. Coefficient values (Beta), standard error (SE), lower confidence level (LCL), and upper confidence level (UCL) for all fixed effects in models with a Δ AICc score < 4 from Table 2: Visits to Sites. Shaded rows represent coefficient values in which the confidence interval does not overlap zero, an indication of the significance of this value.

**Model Rank and Name**	**Fixed Effect**	**Beta**	**SE**	**LCL**	**UCL**
Model Rank = 1 Region + Trial	Region	2.23	0.29	1.66	2.80
	Trial	-1.42	0.22	-1.85	-0.99
Model Rank = 2 Region * Trial	Region	2.08	0.31	1.47	2.69
	Trial	-2.48	1.04	-4.52	-0.44
	Region:Trial	1.14	1.06	-0.94	3.22

Supplementary Table S4. Coefficient values (Beta), standard error (SE), lower confidence level (LCL), and upper confidence level (UCL) for all fixed effects in models with a Δ AICc score < 4 from Table 2: Spatial Response. Shaded rows represent coefficient values in which the confidence interval does not overlap zero, an indication of the significance of this value.

**Model Rank and Name**	**Fixed Effect**	**Beta**	**SE**	**LCL**	**UCL**
Model Rank = 1 Region*Trial+Distance	Region	0.59	0.25	0.10	1.08
	Trial	-2.00	0.74	-3.45	-0.55
	Distance Far	0.89	0.15	0.60	1.18
	Distance On	-0.17	0.18	-0.52	0.18
	Region:Trial	1.58	0.77	0.07	3.09
Model Rank = 2 Region+Trial+Distance	Region	0.89	0.23	0.44	1.34
	Trial	-0.60	0.19	-1.05	-0.15
	Distance Far	0.89	0.15	0.60	1.18
	Distance On	-0.16	0.18	-0.51	0.19

Supplementary Table S5. Coefficient values (Beta), standard error (SE), lower confidence level (LCL), and upper confidence level (UCL) for all fixed effects in models with a ∆ AICc score < 4 from Table 2: Behavioral Response. Shaded rows represent coefficient values in which the confidence interval does not overlap zero, an indication of the significance of this value.

**Model Rank and Name**	**Fixed Effect**	**Beta**	**SE**	**LCL**	**UCL**
Model Rank = 1 Region+Trial+Behavior	Region	0.88	0.32	0.25	1.51
	Trial	-0.59	0.28	-1.14	-0.04
	Behavior-Vigilant	-0.97	0.16	-1.28	-0.66
	Behavior-Comfort	-0.66	0.18	-1.01	-0.31
Model Rank = 2 Region+Behavior	Region	0.67	0.31	0.06	1.28
	Behavior-Vigilant	-0.99	0.16	-1.30	-0.68
	Behavior-Comfort	-0.58	0.18	-0.93	-0.23
